# AST to Platelet Ratio Index (APRI) is an easy-to-use predictor score for cardiovascular risk in metabolic subjects

**DOI:** 10.1038/s41598-021-94277-3

**Published:** 2021-07-21

**Authors:** Carlo De Matteis, Marica Cariello, Giusi Graziano, Stefano Battaglia, Patrizia Suppressa, Giuseppina Piazzolla, Carlo Sabbà, Antonio Moschetta

**Affiliations:** 1 INBB, National Institute for Biostructures and Biosystems, Viale delle Medaglie d’Oro 305, 00136 Rome, Italy; 2 Clinica Medica “Cesare Frugoni”, Department of Interdisciplinary Medicine, Aldo Moro University of Bari, Piazza Giulio Cesare 11, 70124 Bari, Italy; 3 Depatment of Tissues and Organs Transplantation and Cellular Therapies, “Aldo Moro” University of Bari, Bari, Italy

**Keywords:** Metabolic syndrome, Obesity, Liver fibrosis, Non-alcoholic fatty liver disease

## Abstract

Visceral obesity is characterized by a low-grade inflammatory systemic state that contributes to the genesis of non-alcoholic fatty liver disease (NAFLD), frequently associated with liver fibrosis. Non-invasive serum markers have recently emerged as reliable, easy-to-use scores to predict liver fibrosis. NAFLD is often linked to metabolic and cardiovascular risk. Thus, in this cross-sectional study, we investigated in a population of 1225 subjects if AST to Platelet Ratio Index (APRI), one of the non-invasive liver fibrosis serum markers, can predict cardiovascular risk (CVR). APRI has been previously validated as an efficient score to predict liver fibrosis in viral hepatitis patients with a cut-off of 0.5 for fibrosis and 1.5 for cirrhosis. Our study showed that APRI significantly correlates with CVR and determines, when elevated, a significant increase in CVR for both genders, especially females. This spike in CVR, observed when APRI is elevated, is relatively high in patients in the age of 51–65 years, but it is significantly higher in younger and premenopausal women, approaching risk values usually typical of men at the same age. Taken together, our data highlighted the role of APRI as a reliable predictor easy-to-use score for CVR in metabolic patients.

## Introduction

Metabolic Syndrome (MetS) is a systemic condition characterized by a complex spectrum of clinical factors such as visceral obesity, hypertension, and impaired glucose as well as lipid homeostasis^1^. Since 2000, the MetS definition has seen several upgrades and changes, the last being in 2006, when the International Diabetes Federation (IDF) established the following criteria to define the MetS: i.e. waist circumference (WC) > 94 cm in males or > 80 cm in females; Systolic Arterial Pressure (SAP) ≥ 130 mmHg or diastolic blood pressure ≥ 85 mmHg (or drug treatment for hypertension); fasting plasma glucose (FPG) ≥ 100 mg/dL (or drug treatment for elevated glucose); triglycerides (TG) ≥ 150 mg/dL (or drug treatment for elevated triglycerides); high density lipoprotein (HDL) concentration < 40 mg/dL in males or < 50 mg/dL in females (or drug treatment for dyslipidaemia).

Increased values of WC and the contemporary presence of two or more other criteria leads, according to the aforementioned definition, to the diagnosis of MetS^2^. Above all, the EPIC study^3^ highlighted the role of WC as a non-invasive method to predict the presence of excessive visceral adipose tissue and anticipate the risk of death due to cardiovascular events. Indeed, the excess of visceral fat is considered crucial for MetS complications, as it is to determine a major increment in cardiovascular risk^4,5^. Moreover, MetS is characterized by a low-grade inflammatory condition determined by an increased production of adipokines, chemokines and cytokines, as well as an atypical activation of immune cells. Sub-clinical inflammation, along with increased insulin resistance and an array of additional health problems, contributes to the genesis of major complications of MetS: atherosclerotic plaque formation, Non-Alcoholic Fatty Liver Disease (NAFLD) and Non-alcoholic Steato-hepatitis (NASH)^6^.

Although liver biopsy is the gold standard for the NASH diagnosis, non-invasive markers of fibrosis are being incorporated into the routine clinical care of patients with liver disease. The availability of accurate non-invasive tests has allowed to screen large cohorts for significant liver diseases, allowing the assessment of the real burden of the liver disease in the general population.

As of late, non-invasive liver fibrosis scores have been used as systemic inflammation markers^7^ besides different ratios and scores such as neutrophil-to-lymphocyte ratio (NLR)^8,9^, platelet-to-lymphocyte ratio (PLR)^10,11^, lymphocyte-to-monocyte ratio (LMR)^12,13^ and monocyte-to-HDL ratio (MHR)^14^ indexes. Indeed, several studies investigated the role of NAFLD score (NFS), AST to Platelet Ratio Index (APRI score) and FIB-4^15^ in predicting the role of MetS in non-hepatitis patients, investigating why liver failure is linked to cardiovascular diseases, even though the association between all the scores and MetS is still not well established^16,17^.

The AST to Platelet Ratio Index (APRI score) is an easy-to-use score to predict liver fibrosis^18^, such as other noninvasive serum markers (FIB-4 index, FLI, NFS, BARD, Forns index^19^). APRI and FIB-4 can easily be calculated as fast, low cost and practice alternative to other tests, especially compared to liver biopsy and further invasive tests. APRI is still often used to evaluate the level of liver fibrosis in patients affected by hepatitis B or C and combines commonly obtained laboratory tests, whereas FIB-4 integrates other factors such as age to its formula.

Given the relevance of systemic inflammation in NAFLD, often associated with MetS, in the present study we analysed the potential role of APRI score in predicting the cardiovascular risk (CVR) using the Framingham Heart Study^20^ in metabolic subjects.

## Results

### Clinical characterization of the study population

The baseline characteristics of the study population are shown in Table 1 and Supplementary Table 1. We enrolled 540 MetS patients (MetS YES) and 685 non-metabolic subjects (MetS NO). Compared to non-metabolic subjects, MetS patients exhibited increased waist circumferences (WC), blood pressure, BMI, glycemia, HbA1c, triglycerides (TG), decreased HDL and increased levels of inflammation markers (WBC, neutrophils, Hs-CRP, ESR). Platelet count was not significantly different between the two groups. Liver markers such as aspartate transaminase (AST), alanine transaminase (ALT), alkaline phosphatase (ALP) and gamma-glutamyl transferase (GGT) were found significantly higher in MetS patients. Compared to non-metabolic subjects, levels of ferritin were higher in MetS patients, while iron and creatinine concentration was lower in this group. Considering non-invasive indexes, MetS patients presented increased values of CVR, APRI and FIB-4 score (Table 1 and Supplementary Table 1).

**Table 1:** Clinical characterization of the study population.

Clinical variable	MetS NO	MetS YES	p-value
n (M:F)	685 (336:349)	540 (287:263)	–
Weight (kg)	76.89 ± 0.71	83.21 ± 1.32	< 0.05
Waist circumference (cm)	95.9 ± 0.91	105.9 ± 1.21	< 0.05
BMI (kg/m^2^)	26.9 ± 0.39	30 ± 0.39	< 0.05
Sistolic blood pressure (mmHg)	124.0 ± 0.28	134 ± 0.99	< 0.05
Diastolic blood pressure (mmHg)	76.9 ± 0.27	81.4 ± 0.73	< 0.05
Platelet count (10^6^/μL)	228.21 ± 3.46	193.7 ± 3.48	NS
Hemoglobin (g/dl)	13.99 ± 0.02	14.15 ± 0.02	NS
WBC (10^3^/µl)	5.99 ± 0.04	6.99 ± 0.03	< 0.05
Monocytes (%)	6.54 ± 0.02	6.51 ± 0.04	NS
Lymphocytes (%)	32.38 ± 0.60	32.02 ± 0.43	NS
Neutrophils (%)	57.01 ± 0.22	58.26 ± 0.03	NS
Basophils (%)	0.53 ± 0.01	0.55 ± 0.02	NS
Eosinophils (%)	2.79 ± 0.07	2.79 ± 0.07	NS
Glucose (mg/dl)	91.94 ± 1.29	121.4 ± 2.02	< 0.05
HbA1c (mmol/mol)	37.5 ± 0.43	43.23 ± 0.83	< 0.05
Total cholesterol (mg/dl)	173 ± 3.23	172.29 ± 2.93	NS
HDL-c (mg/dl)	58.42 ± 1.23	44.32 ± 0.73	< 0.05
LDL-c (mg/dl)	102.43 ± 1.32	101.23 ± 1.32	NS
TG (mg/dl)	95.43 ± 1.32	158.32 ± 3.21	< 0.05
AST (U/I)	27.32 ± 0.39	29.32 ± 0.22	NS
ALT (U/I)	32.2 ± 0.51	38.32 ± 1.49	< 0.05
ALP (U/I)	70.21 ± 1.32	72.12 ± 1.32	< 0.05
GGT (U/I)	34.32 ± 1.83	51.22 ± 3.22	< 0.05
Ferritin (ng/ml)	121.32 ± 8.32	134.32 ± 11.48	NS
Iron (µg/dl)	83.21 ± 3.42	78.32 ± 3.22	NS
Creatinine (mg/dl)	0.84 ± 0.02	0.86 ± 0.01	NS
Uric acid (mg/dl)	48.67 ± 0.19	58.08 ± 0.35	NS
Total protein (g/dl)	7.24 ± 0.03	7.32 ± 0.03	NS
Albumin (g/dl)	4.52 ± 0.21	4.99 ± 0.22	NS
ESR (mm/h)	14 ± 1.7	21.21 ± 1.24	< 0.05
Hs-CRP (mg/l)	3.71 ± 0.34	4.43 ± 0.86	NS
TSH (mUI/L)	1.83 ± 0.35	2.09 ± 0.21	NS
FT3 (pg/ml)	2.80 ± 0.12	2.54 ± 0.02	NS
FT4 (ng/dl)	1 ± 0.21	1.02 ± 0.12	NS
Ab anti TG (UI/ml)	42.32 ± 15.03	65.23 ± 32.93	NS
Ab anti TPO (UI/ml)	301.12 ± 133.32	183 ± 132.74	< 0.05
Cardiovascular risk (Framingham)	13.43 ± 1.43	32.91 ± 1.91	< 0.05
FIB-4 score	1.3 ± 0.09	1.84 ± 0.02	< 0.01
APRI score	0.3 ± 0.01	0.62 ± 0.02	< 0.01

Data are age-adjusted and presented as mean ± SEM (standard error of the mean).

*BMI* Body Mass Index, *WC* waist circumference, *SBP* systolic blood pressure, *DBP* diastolic blood pressure, *HbA1c* glycosylated hemoglobin, *TC* total cholesterol, *HDL-C* high-density lipoprotein cholesterol, *LDL-C* low-density lipoprotein cholesterol, *TG* triglyceride, *AST* aspartate transaminase, *ALT* alanine transaminase, *ALP* alkaline phosphatase, *GGT* gamma-glutamyltransferase, *ESR* erythrocyte sedimentation rate, *Hs-CRP* high-sensitivity C reactive protein.

### Association of APRI score with MetS criteria

**Figure 1:**
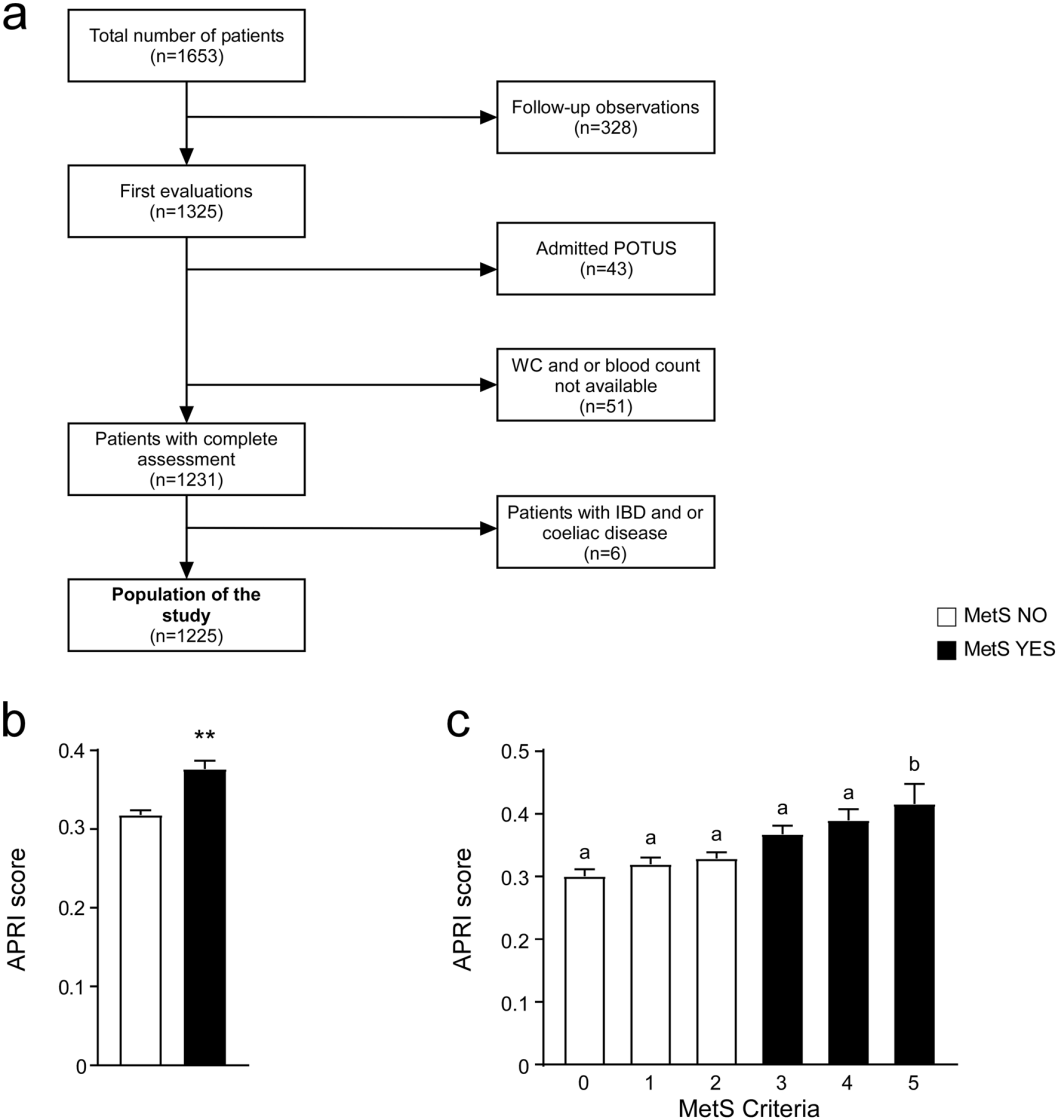
Flowchart of study population and graphic representation of APRI score values. (**a**) Flowchart of the study population. (**b**) T-Test comparison of entire study population. Subjects categorized based on Metabolic Syndrome (MetS) diagnosis; non-metabolic (MetS NO) subjects = n.685, MetS (MetS YES) patients = n.540. Data is presented as mean ± SEM; **p < 0.01. (**c**) Comparison of APRI levels. Subjects were categorized based on positivity for MetS criteria. 0 criteria = n.186, 1 criterion = n.240, 2 criteria = n.259, 3 criteria = n.283, 4 criteria = n.171, 5 criteria = n.86. Data is presented as mean ± SEM. Comparison of groups was performed using one-way ANOVA test followed by Bonferroni’s post-hoc test. Data from groups sharing the same lowercase letter were not significantly different, whereas data from groups with different case letter were significantly different (p < 0.05).

To evaluate the link between APRI score and metabolic conditions, we compared APRI levels in non-metabolic and MetS patients, showing that MetS patients have significantly higher APRI, when compared to non-metabolic subjects (Fig. 1b). To further characterise our study population, we considered MetS criteria observing that APRI score significantly increased with the number of MetS parameters, especially in MetS patients (Fig. 1c).

**Figure 2:**
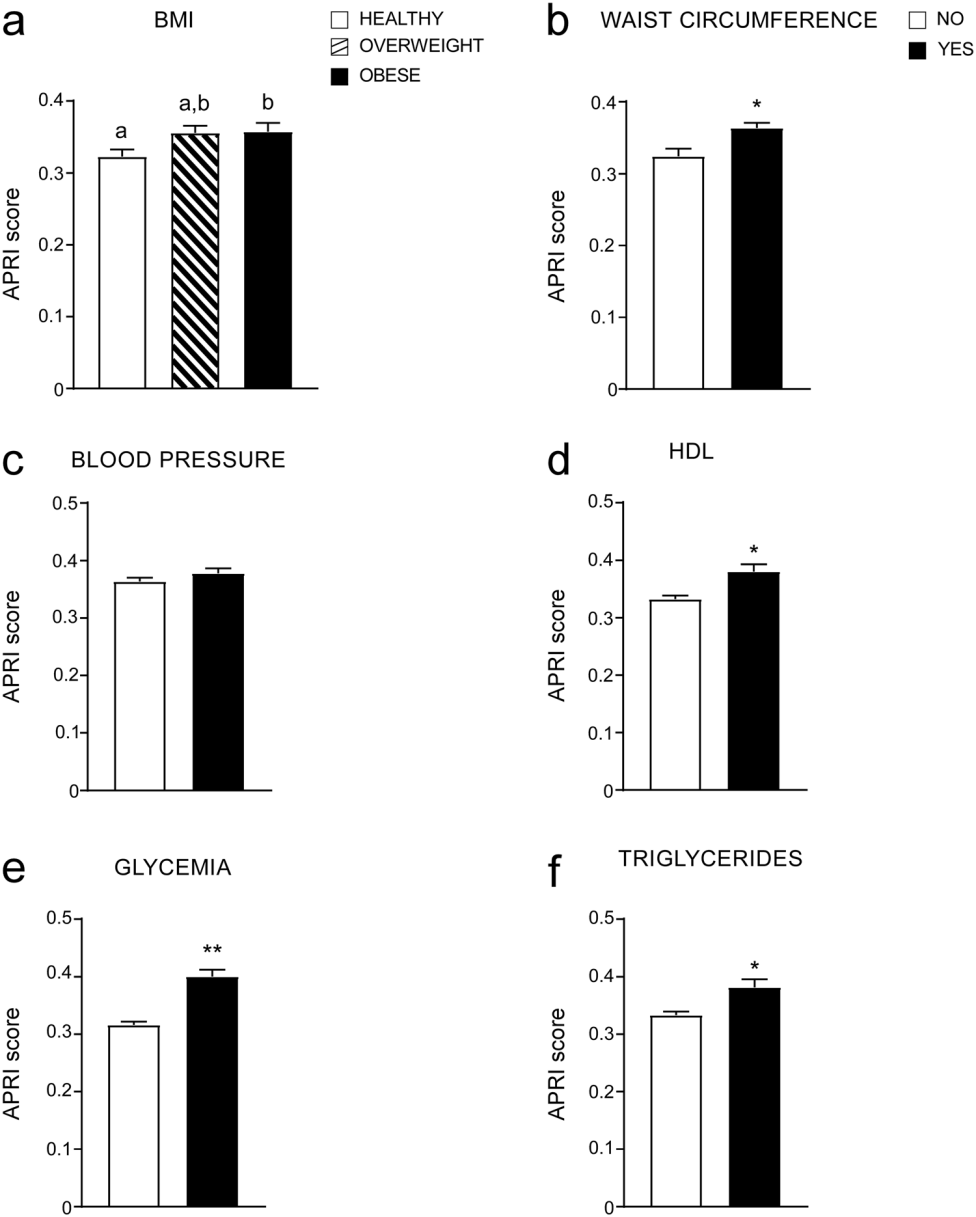
APRI score levels analyzed by single MetS criterion and BMI cut-offs. (**a**) Comparison of APRI values according to BMI definition. Subjects were divided in three different categories. Healthy = n. 412, Overweight = n. 421, Obese = n.305. Data is presented as mean ± SEM. Comparison of groups was performed using one-way ANOVA test followed by Bonferroni’s post-hoc test. Data from groups sharing the same lowercase letters were not significantly different, whereas data from groups with different case letters were significantly different (p < 0.05). (**b**–**f**) T-Test comparison of APRI values in subjects divided by positivity for each MetS criterion according to IDF definition. Data is presented as mean ± SEM. *p < 0.05, **p < 0.01.

Given the role of MetS in defining CVR, we analysed the relation between BMI, MetS and APRI, comparing APRI levels in normal, overweight and obese patients (according to the BMI definition^21^), showing that APRI increased in overweight patients as well as in obese subjects compared to healthy subjects (Fig. 2a). Furthermore, we observed a significant up-regulation of APRI levels in patients with elevated WC, low HDL levels, high glycemia and trygliceride levels (MetS criteria) (Fig. 2b,d–f). No differences in APRI levels were found in patients with increased blood pressure (Fig. 2c). Interestingly, considering all criteria, glycemia stands as the major determinant of elevated APRI levels. These data confirm the relation between APRI and MetS, showing that each criteria, if positive for MetS, determines an increment of APRI score.

To characterize the link between APRI and CVR, we further divided our population between non-metabolic subjects and MetS according to cut-off value for APRI (see material and method section). Both non-metabolic subjects and MetS groups with APRI > 0.5 did not present significant age difference compared to non-metabolic and MetS groups with APRI < 0.5, respectively. Furthermore, liver markers (AST, ALT, ALP, GGT) were significantly higher in non-metabolic subjects and MetS patients with APRI > 0.5 compared to non-metabolic and MetS groups with APRI < 0.5. The statistical comparison of CVR confirmed that there is a significant difference when APRI > 0.5 in the non-metabolic and MetS groups, yet especially in non-metabolic subjects with APRI > 0.5, it is particularly important to note that the CVR reaches the same level as in MetS with APRI < 0.5 (Table 2 and Supplementary Table 2). These data highlight the role of elevated APRI as a non-invasive predictor for metabolic disorders. Both non-metabolic and MetS subjects with APRI > 0.5 showed major increase in CVR compared to reciprocal cohort with APRI < 0.5, as well as elevated values for MetS risk factors.

**Table 2:** Clinical comparison between non-metabolic and MetS subjects with normal and elevated APRI score in the study population.

Clinical variable	MetS NO		MetS YES		p-value
	APRI < 0.5	APRI > 0.5	APRI < 0.5	APRI > 0.5	
n (M:F)	587 (283:304)	98 (53:45)	406 (216:200)	134 (71:63)	–
Weight (kg)	73.43 ± 0.70	78.93 ± 2.44	84.04 ± 0.81^a^	87.21 ± 2.01	< 0.05
Waist circumference (cm)	91.99 ± 0.61	95.24 ± 1.68	106.66 ± 0.61^a,b^	106.54 ± 1.32^a^	< 0.01
BMI (kg/m^2^)	25.62 ± 0.39	26.95 ± 1.23	30.62 ± 0.31	30.59 ± 1.43	NS
Sistolic blood pressure (mmHg)	120.94 ± 0.74	124.71 ± 2.09	134.61 ± 0.90^a^	134.41 ± 1.51^a^	< 0.05
Diastolic blood pressure (mmHg)	76.15 ± 0.32	76.13 ± 1.10	80.77 ± 0.31	81.55 ± 1.04	NS
Platelet count (10^6^/μL)	245.11 ± 2.76	172.74 ± 4.99^a,c^	243.31 ± 2.31	179.94 ± 4.93^a,c^	< 0.05
Hemoglobin (g/dl)	13.91 ± 0.12	14.10 ± 0.31	13.81 ± 1.31	14.10 ± 0.13	NS
WBC (103/µl)	6.03 ± 0.08	5.60 ± 0.28	7.04 ± 1.83	6.30 ± 0.16	NS
Monocytes (%)	6.24 ± 0.07	6.81 ± 0.34	6.21 ± 0.04	6.78 ± 0.19	NS
Lymphocytes (%)	33.01 ± 0.38	32.89 ± 1	30.37 ± 0.38	32.98 ± 0.94	NS
Neutrophils (%)	57.51 ± 0.31	56.54 ± 1.21	59.42 ± 0.29	57.07 ± 0.94	NS
Basophils (%)	0.53 ± 0.03	0.6 ± 0.04	0.53 ± 0.02	0.56 ± 0.03	NS
Eosinophils (%)	2.73 ± 0.11	2.82 ± 0.32	2.71 ± 0.99	2.78 ± 0.21	NS
Glucose (mg/dl)	88.27 ± 0.81	92.30 ± 2.89	116 ± 2.82^a,b^	120.61 ± 3.94^a,b^	< 0.05
HbA1c (mmol/mol)	37.47 ± 0.30	37.89 ± 1.51	47.82 ± 0.94^a,b^	46.83 ± 1.41^a,b^	< 0.05
Total cholesterol (mg/dl)	189.04 ± 1.09	174.38 ± 4.31	175.12 ± 1.40	181.15 ± 3.99	NS
HDL-c (mg/dl)	60.65 ± 0.51	54.69 ± 2.31	47.01 ± 0.71^a,b^	44.44 ± 1.32^a,b^	< 0.01
LDL-c (mg/dl)	110.42 ± 1.21	99.5 ± 4.40	99.31 ± 1.14	99.32 ± 3.42	NS
TG (mg/dl)	91.54 ± 1.32	95.64 ± 3.84	158.13 ± 4.31^a,b^	165 ± 7.99^a,b^	< 0.05
AST (U/I)	20.06 ± 0.21	34.73 ± 1.03^a^	20.54 ± 0.31	41.88 ± 1.52^a,c^	< 0.01
ALT (U/I)	26.35 ± 0.37	43.86 ± 2.96^a,c^	29.79 ± 1.02	58.34 ± 3.25^a,c^	< 0.05
ALP (U/I)	66.47 ± 1.42	75.19 ± 2.98	72.12 ± 1.42	73.36 ± 2.52	NS
GGT (U/I)	27.11 ± 0.88	47 ± 4.93^a^	37.32 ± 1.32	62.17 ± 5.31^a,c^	< 0.01
Ferritin (ng/ml)	96.32 ± 3.98	159.29 ± 13.99^a,c^	114.32 ± 9.48^a^	180.73 ± 18.33^a,c^	< 0.05
Iron (µg/dl)	84.21 ± 2.50	93.98 ± 7.12	86.32 ± 3.29	75.53 ± 4.23b	NS
Creatinine (mg/dl)	0.80 ± 1.49	0.86 ± 0.02	0.91 ± 1.99	0.87 ± 2.01	NS
Uric acid (mg/dl)	53.83 ± 0.18	57.99 ± 0.40	58.02 ± 0.31	60.33 ± 0.91^a^	NS
Total protein (g/dl)	7.24 ± 1.40	7.22 ± 4.81	7.30 ± 4.32	7.41 ± 5.01	NS
Albumin (g/dl)	4.63 ± 0.84	4.43 ± 2.85	5.13 ± 0.59	4.37 ± 0.15	NS
ESR (mm/h)	14.02 ± 0.48	14.24 ± 1.73	21.32 ± 1.11^a,b^	18.91 ± 1.94	NS
Hs-CRP (mg/l)	3.68 ± 0.17	3.76 ± 0.32	4.93 ± 0.42	4.50 ± 0.36	NS
TSH (mUI/L)	1.84 ± 0.10	2.02 ± 0.17	2.09 ± 0.63	1.96 ± 0.23	NS
FT3 (pg/ml)	2.82 ± 1.83	2.82 ± 6.01	2.76 ± 2.96	2.80 ± 5.83	NS
FT4 (ng/dl)	1.01 ± 1.12	0.99 ± 0.84	1.06 ± 1.45	1.07 ± 2.99	NS
Ab anti TG (UI/ml)	42.73 ± 12.01	42.69 ± 27.31	96 ± 47.86	15.36 ± 2.44	NS
Ab anti TPO (UI/ml)	441.71 ± 160.54	38.83 ± 13.93	259.96 ± 214.93	19.10 ± 2.86	NS
Cardiovascular risk (Framingham)	9.93 ± 0.83	21.95 ± 1.54^a^	24.51 ± 0.91^a^	39.97 ± 2.01^a,b,c^	< 0.01
FIB-4 score	1.13 ± 0.14	1.58 ± 0.16	1.04 ± 0.03	2.05 ± 0.21^a,c^	< 0.05
APRI score	0.28 ± 0.06	0.67 ± 0.04^a,c^	0.28 ± 0.02	0.84 ± 0.09^a,c^	< 0.01

Data are age-adjusted and presented as mean ± SEM (standard error of the mean).

*BMI* Body Mass Index, *WC* waist circumference, *SBP* systolic blood pressure, *DBP* diastolic blood pressure, *TC* total cholesterol, *TG* triglyceride, *HDL-C* high-density lipoprotein cholesterol, *LDL-C* low-density lipoprotein cholesterol, *HbA1c* glycosylated hemoglobin, *Hs-CRP* high-sensitivity C reactive protein, *ESR* erythrocyte sedimentation rate, *GGT* gamma-glutamyltransferase, *AST* aspartate transaminase, *ALT* alanine transaminase, *ALP* alkaline phosphatase.

^a^Indicates statistical significance compared to MetS NO with APRI < 0.5 group.

^b^Indicates statistical significance compared to MetS NO with APRI > 0.5 group.

^c^Indicates statistical significance to MetS YES with APRI < 0.5 group.

Overall, these data highlighted the role of elevated APRI to predict a significant increment in CVR in both non-metabolic and metabolic subjects.

### Correlation between APRI score and CVR

**Figure 3:**
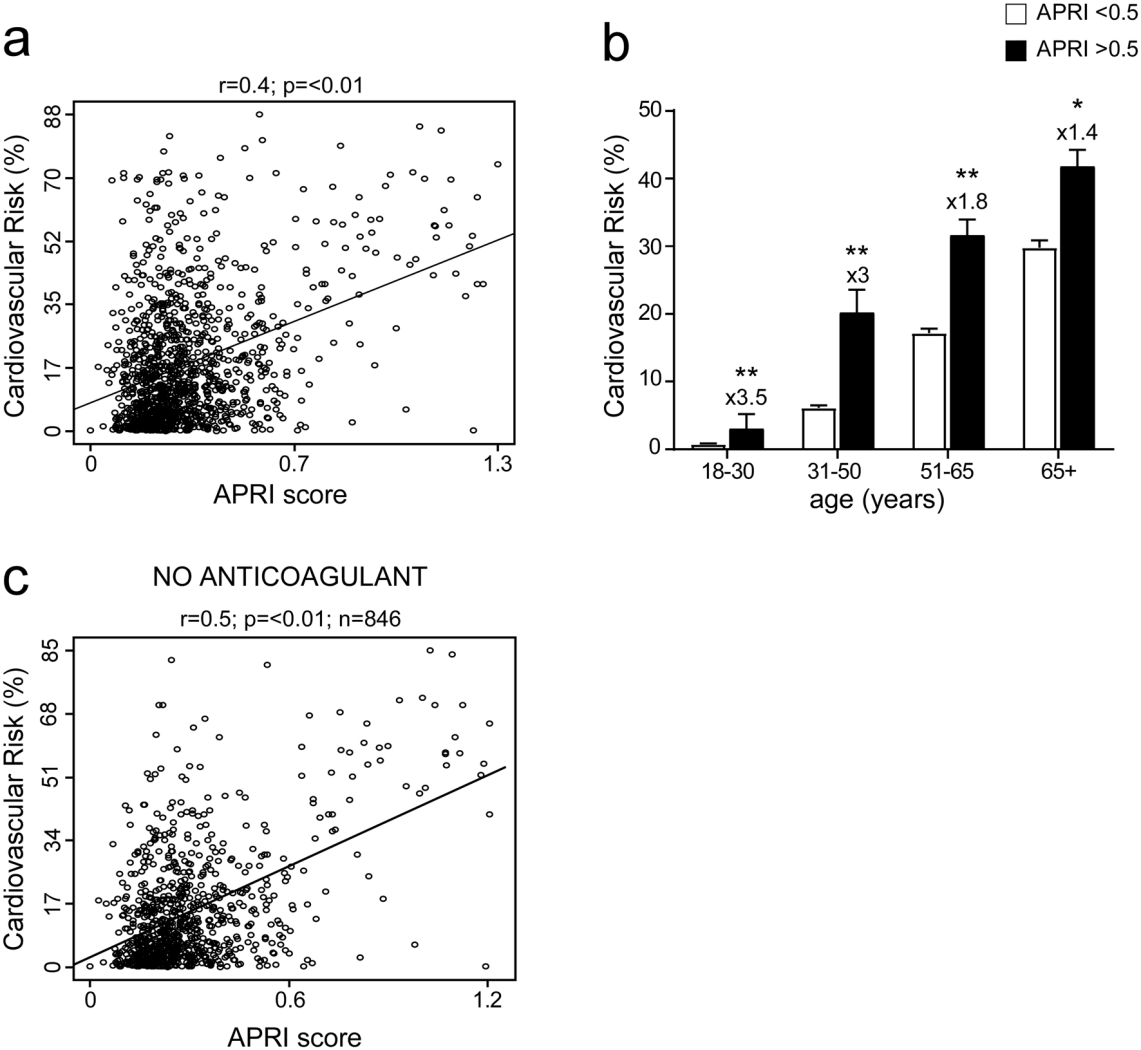
Correlation between APRI score and cardiovascular risk in general population and classification by age. (**a**) Correlation analysis of APRI and cardiovascular risk (CVR) in the entire study population. (**b**) Classification of entire study population in age ranges; subjects were divided by APRI levels with a cut-off of 0.5 to identify elevated APRI. Data is presented as mean ± SEM. Statistical significance was assessed by Student T-test (*p < 0.05, **p < 0.01). Multipliers are shown to identify difference in CVR in subjects with APRI > 0.5 compared to subjects with APRI < 0.5 of the same age. (**c**) Correlation of APRI and CVR after removal of patients ongoing anticoagulant or antiplatelet treatment.

To analyse the relation between APRI and CVR, we calculated the correlation of the two variables in the entire study population of 1142 patients, including the ones who took drugs and other medications that might affect the platelet count and APRI itself. We found a significant and positive correlation of APRI with CVR (Fig. 3a).

Furthermore, we divided our study population to identify four different age ranges. This was necessary to identify if an age increase could determine the relation between APRI and CVR: results showed that in all ranges a pathologic APRI relates to an increased CVR, especially in younger patients, between the age of 18 and 50 years old. (Fig. 3b).

To better characterize our study population, we then excluded from the same analysis patients who took anticoagulant or antiplatelet drugs, which may alter the APRI value, decreasing the study population to 846 patients between non-metabolic subjects and MetS patients (439 and 407 respectively). Results of the correlation showed high statistical relevance (p < 0.01) in MetS patients compared to non-metabolic subjects (Fig. 3c).

**Figure 4:**
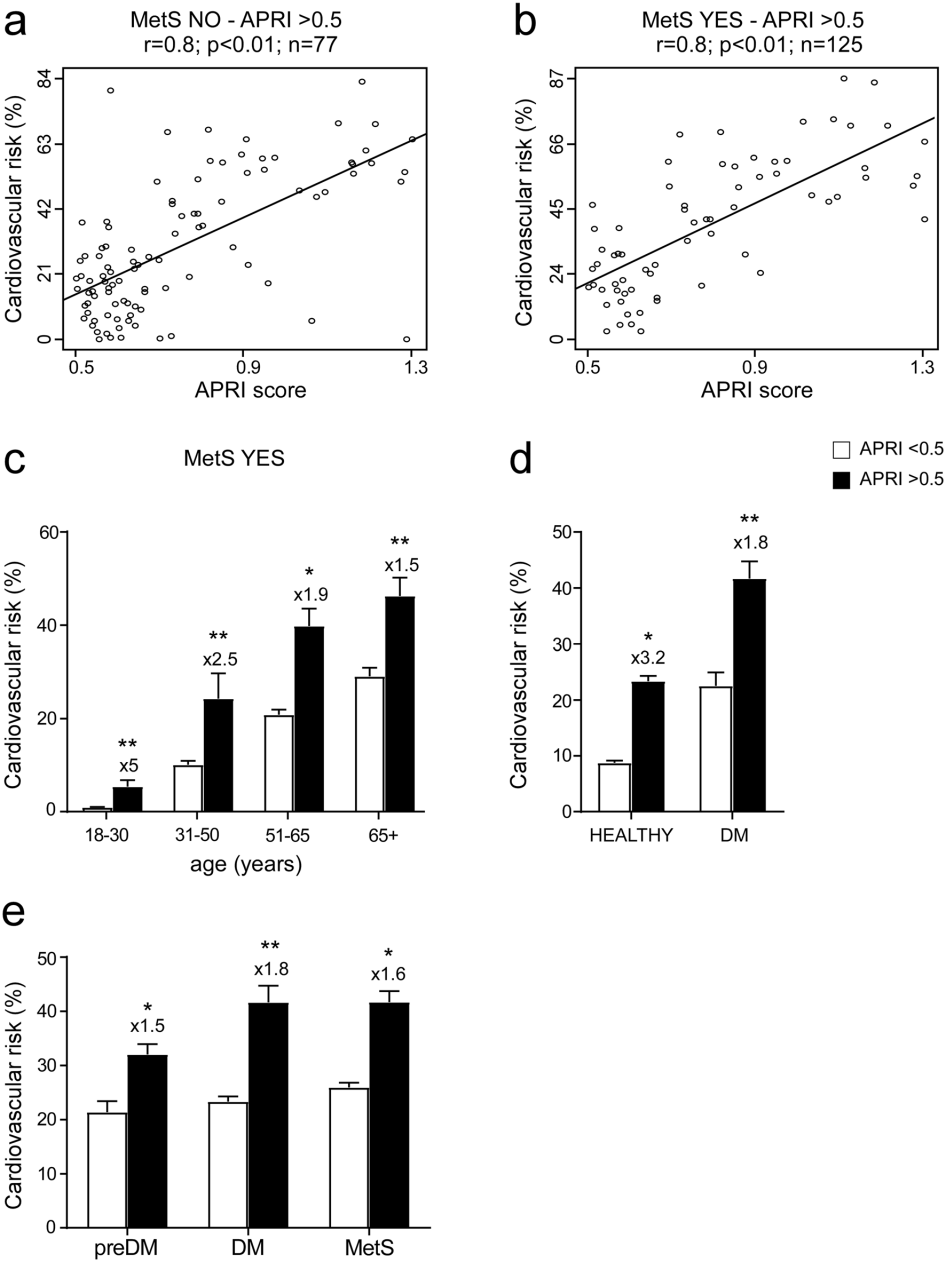
Correlation between APRI > 0.5 and cardiovascular risk in non-metabolic and metabolic subjects, classification by age and comparison in prediabetes and Diabetes Mellitus subjects. (**a**,**b**) Correlation analysis of APRI and cardiovascular risk (CVR) in non-metabolical (MetS NO) and MetS (MetS YES) subjects with APRI > 0.5. (**c**) Classification of MetS subjects in age ranges; subjects were divided by APRI levels with a cut-off of 0.5 to identify elevated APRI. Data is presented as mean ± SEM. Statistical significance was assessed by Student T-test (**p < 0.01). Multipliers are shown to identify difference in CVR in subjects with APRI > 0.5 compared to subjects with APRI < 0.5 of the same age. (**d**) Comparison of CVR in healthy and Diabetes Mellitus (DM) subjects divided by APRI levels. Data is presented as mean ± SEM. Statistical significance was assessed by Student T-test (*p < 0.05, **p < 0.01). Multipliers are shown to identify difference in CVR in subjects with APRI > 0.5 compared to subjects with APRI < 0.5 of the same age. (**e**) Comparison of CVR in PreDiabetes (preDM), Diabetes Mellitus (DM) and Metabolic (MetS) subjects divided by APRI levels. Data is presented as mean ± SEM. Statistical significance was assessed by Student T-test (*p < 0.05, **p < 0.01). Multipliers are shown to identify difference in CVR in subjects with APRI > 0.5 compared to subjects with APRI < 0.5 of the same group.

Furthermore, to evaluate the link between APRI and CVR, we divided our cohort in non-metabolic and MetS subjects with APRI > 0.5 (77 non-metabolic and 125 MetS subjects). We observed a strong correlation (r = 0.8) between APRI and CVR in both non-metabolic and MetS subjects with APRI > 0.5 (Fig. 4a,b), demonstrating that high levels of APRI determine a major increase in CVR in both groups.

Furthermore, to analyse the correlation between APRI > 0.5 and MetS we divided our cohort using the same age ranges previously defined (Fig. 3). We found that APRI > 0.5 determines an increment in CVR in all age ranges (Fig. 4c). This is particularly evident when comparing CVR in MetS with APRI > 0.5 in 18–30 years old group, who have shown a five-fold CVR to MetS with APRI < 0.5, as well as in the range between age 31 and 50 years old, in which there is a more than double risk (Fig. 4c). To further investigate if APRI determines the same increment in CVR even in different cohorts, we compared the level of CVR in healthy and diabetic (DM) patients: data showed that even in the diabetic group, APRI > 0.5 strongly influenced the outcome of the analysis, affecting the CVR which is found to be higher in subjects with elevated APRI than in diabetic patients (Fig. 4d). Therefore, to better comprehend the role of APRI > 0.5, we compared the level of CVR in prediabetes subjects (preDM), DM and MetS patients: data showed that in each group, APRI > 0.5 constantly determines a major increase in CVR (Fig. 4e).

Taken together, our data showed that APRI correlates with MetS criteria and is significantly related with CVR in preDM subjects, as well as in MetS and DM patients.

### Gender difference in APRI and CVR correlation

**Figure 5:**
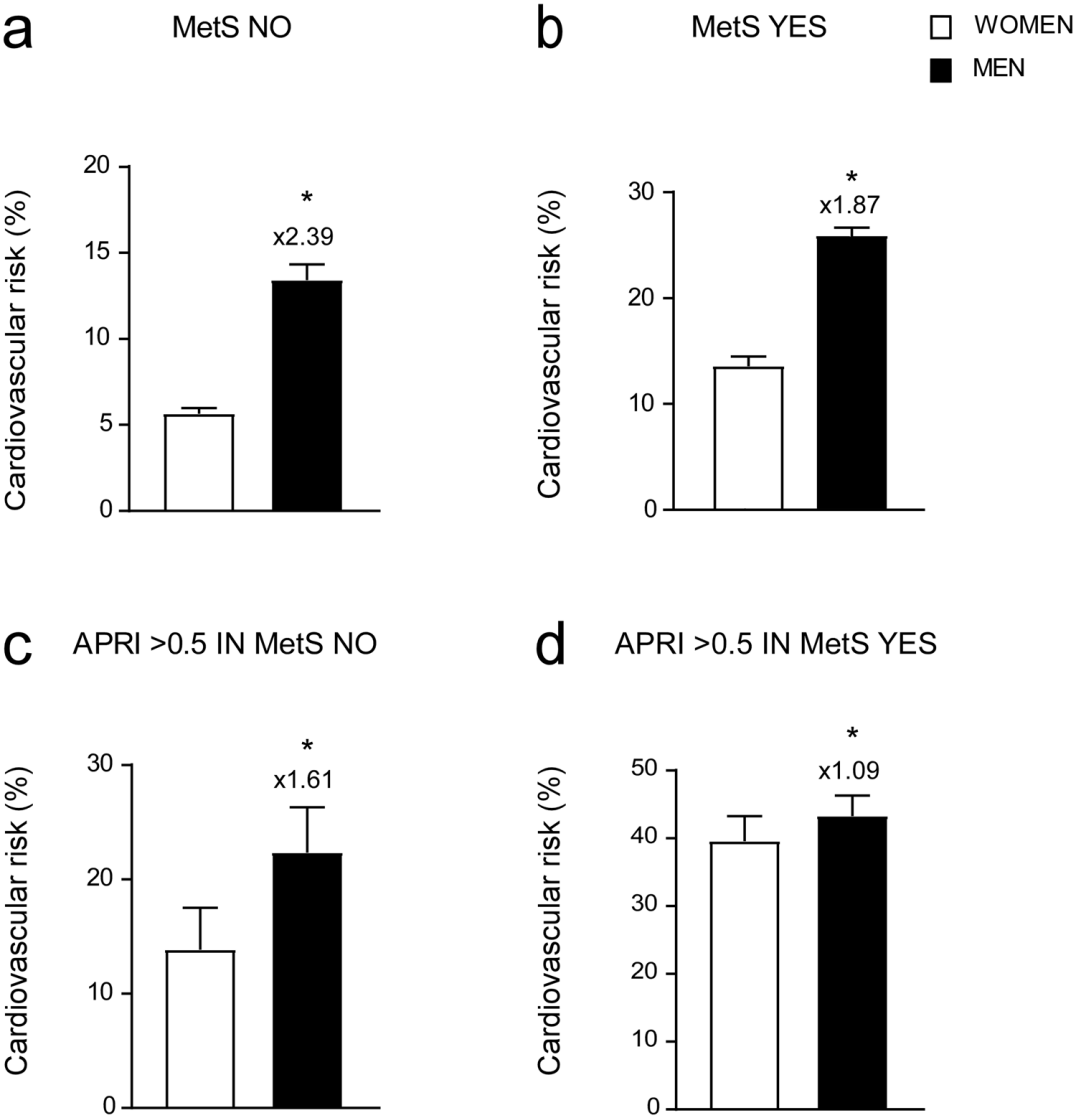
Gender-based comparison of cardiovascular risk in different groups. (**a**–**d**) T-Test comparison of CVR in non-metabolic (MetS NO) (**a**), MetS (MetS YES) (**b**), non-metabolic (MetS NO) with APRI > 0.5 (**c**) and MetS (MetS YES) subjects with APRI > 0.5 (**d**). Subjects were divided based on gender. Data is presented as mean ± SEM. Statistical significance was assessed by Student T-test (**p < 0.01). Multipliers are shown to identify difference in CVR in males compared to females.

To further characterize the role of APRI in defining CVR in our study population, we proceeded to divide our cohort of patients by gender. We broke down the same groups studied before (non-metabolic, MetS, non-metabolic and MetS subjects with APRI > 0.5). Our data showed that, in non-metabolic subjects, men presented more than double the CVR compared to women, but this difference attenuated in MetS patients (Fig. 5a,b). Furthermore, we analysed non-metabolic and MetS subjects with APRI > 0.5, observing that the gap in CVR between men and women narrows as the level of APRI increases, especially, in patients with a diagnosis of MetS (Fig. 5c,d). Interestingly, we showed that CVR in MetS women with APRI > 0.5 substantially reaches the same value in the group of MetS men with APRI > 0.5. (Fig. 5d).

**Figure 6:**
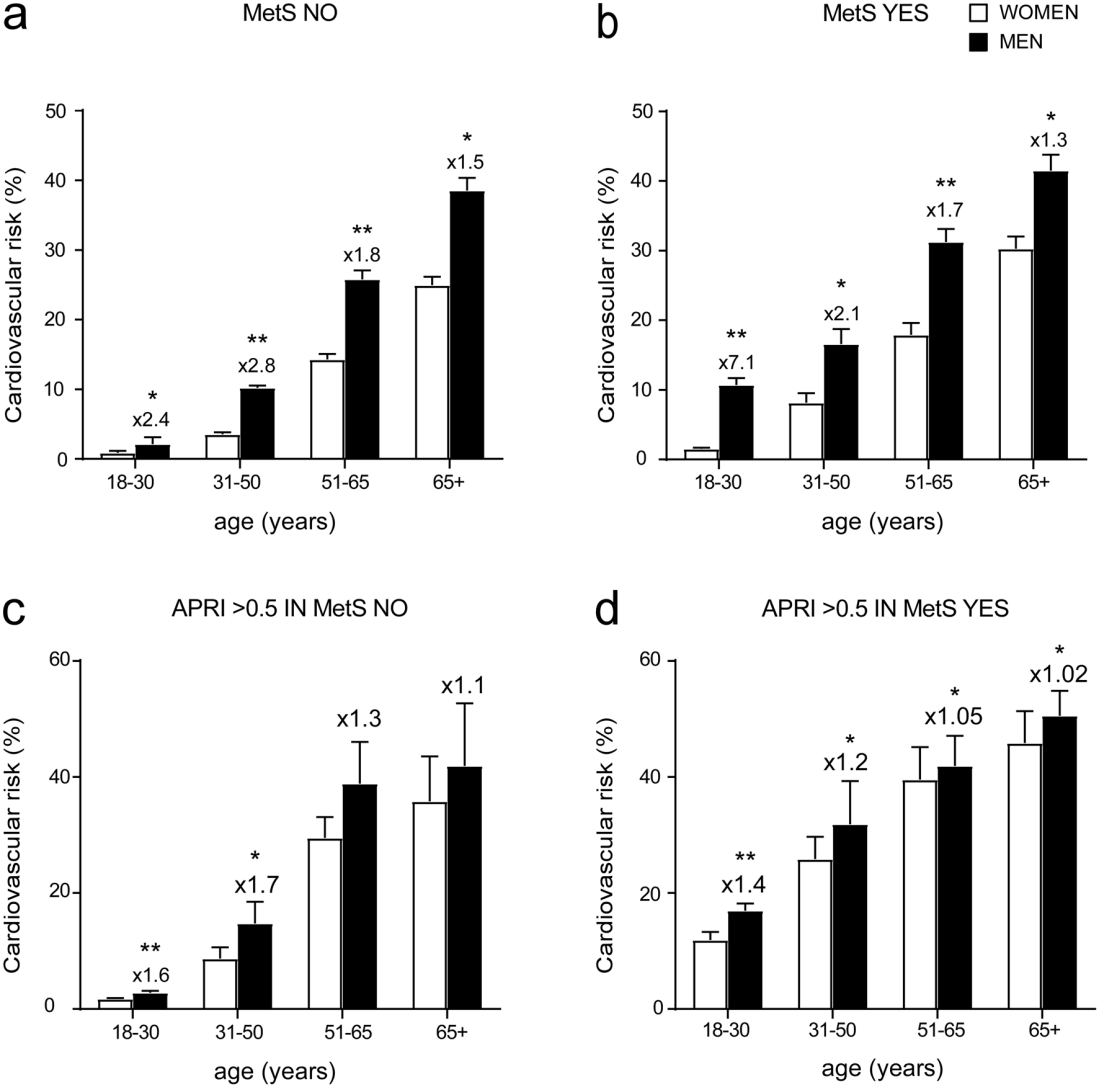
Cardiovascular risk analysis by gender and age. (**a**–**d**) T-Test comparison of CVR in non-metabolic (MetS NO) subjects (**a**), MetS (MetS YES) subjects (**b**), non-metabolic (MetS NO) subjects with APRI > 0.5 (**c**) and MetS (MetS YES) subjects with APRI > 0.5 (**d**). Subjects were divided based on gender and age. Data is presented as mean ± SEM. Statistical significance was assessed by Student T-test (*p < 0.05, **p < 0.01).. Multipliers are shown to identify difference in CVR in males compared to females.

Indeed, to better characterize the correlation between APRI and CVR in gender-based population we analysed in the above-mentioned groups the gender difference between patients with normal and pathologic APRI in the four different age ranges. In non-metabolic subjects, CVR is higher in men, especially in the age ranges 18–30 and 31–50, where it is more than double compared to women of the same age (Fig. 6a). In MetS group, we detected a larger gap between the two genders in the age range 18–30 (Fig. 6b). In the non-metabolic group with APRI > 0.5 we did not observe significant gender-differences at 18–30, 51–65 and 65 + ranges but in 31–50 range we found a significant up-regulation of CVR in men compared to women (Fig. 6c). Interestingly, MetS patients with APRI > 0.5 showed significantly higher CVR in both genders, (CVR = 30.75 ± 3.09 for women and 35.28 ± 4.05 for men) compared to non-metabolic subjects with APRI > 0.5 (CVR = 18.90 ± 3.35 for women and 24.55 ± 5.51 for men) and MetS patients with APRI < 0.5 (CVR = 14.45 ± 1.27 for women and 24.98 ± 1.84 for men). Both genders exhibited increased CVR compared to groups with APRI < 0.5, MetS patients included, but this is particularly evident for women, who reached the same level of CVR of men (Fig. 6d).

**Figure 7:**
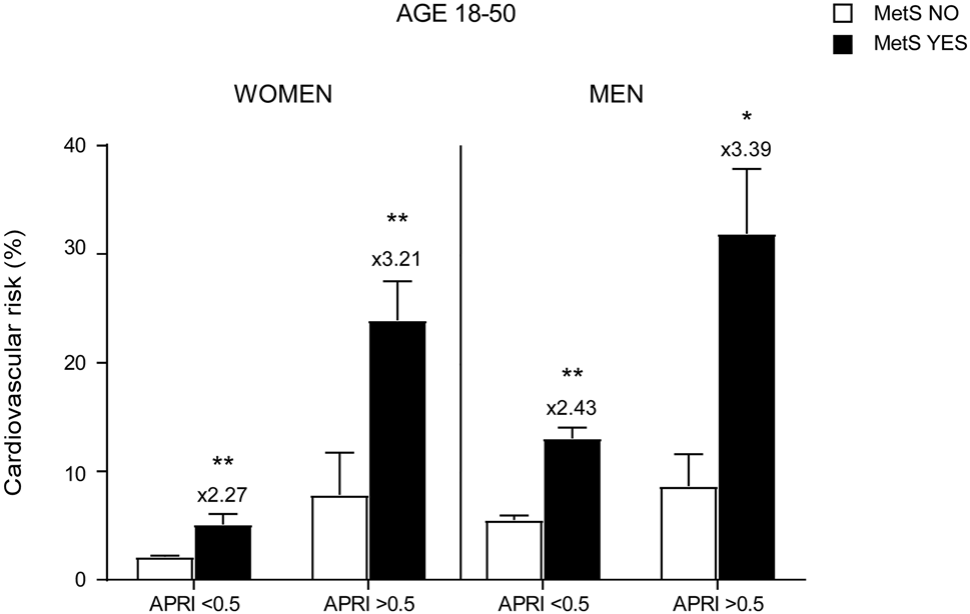
Characterization of cardiovascular risk in adults age 18–50 based on gender and APRI score levels. T-Test analysis of CVR in females and males age 18–50. Subjects were divided based on MetS diagnosis and APRI levels. Data is presented as mean ± SEM. Statistical significance was assessed by Student T-test (*p < 0.05, **p < 0.01). Multipliers are shown to identify difference in CVR in males compared to females.

To further evaluate the capacity of pathologic APRI to determine an increase in CVR, especially in young and under 50 years old patients, we specifically analysed the age range between 18 and 50 years old, also dividing our groups by gender. Results showed that when APRI > 0.5 there is a significant increment in CVR in both genders and both conditions (non-metabolic and MetS subjects) (Fig. 7). Furthermore, a major growth is found in MetS with elevated APRI without gender-difference, but that is particularly evident in women affected by MetS, whom have almost the same CVR as men of the same age and condition (24.01 ± 3.39 in women, versus 30.81 ± 5.95 in men). It is also notable that even in the non-metabolic group, APRI > 0.5 determines a higher CVR if compared to MetS patients with APRI < 0.5 (Fig. 7).

Overall, these data demonstrated that elevated APRI can predict an increase in CVR in both genders, especially in young and premenopausal females.

## Discussion

MetS is a clinical condition characterized by several risk factors for heart disease, diabetes, visceral obesity and low-grade chronic inflammatory status. MetS is part of a multisystem condition with multiple associations and each condition contributes to increment CVR: diastolic dysfunction^22,23^, increased carotid intima media thickness (cIMT)^24^, increased oxidative stress^25,26^, altered lipid profile^27,28^ and systemic inflammation are among the most important pathophysiological mechanisms responsible for increase CVR and cardiovascular disease (CVD) in MetS patients. Currently, the link in MetS patients and CVD development is still not completely understood. Numerous studies have underlined the role of chronic inflammation in MetS^29^, as well as its consequences on cardiovascular health. Nonetheless, the chronic inflammatory state plays a key role in the development of organ fibrosis, with clinical conditions such as NASH and NAFLD as primary consequences. Still, the role of NAFLD as an independent factor for CVD is widely debated. While several studies observed an increased mortality for CVD in NAFLD patients^30,31^, others did not confirm this connection^32,33^, especially with long-term follow up. The most recent EASL-EASD-EASO guidelines regarding NAFLD^34^ have included a comprehensive lifestyle approach, whilst it must be pointed that no specific drugs have been indicated as gold standard for treatment of NASH or NAFLD. Moreover, Tripodi et al. demonstrated^35^ that CVR, as well as procoagulant factors, were increased in NAFLD patients compared to controls^36,37^. In this scenario, to test a large group of patients within the global population to identify potential NAFLD patients, non-invasive serum markers emerge as a low-cost^38^ and ready to use alternative to more invasive, operator-dependant procedures such as liver biopsy, which is recognized as the gold standard^39^ for liver fibrosis staging. Non-invasive serum markers are as well optimal to predict CVR^40^ considering the crucial role played by the liver in lipid and glucose homeostasis^41^. The relation network between the visceral and subcutaneous adipose tissue, gut, muscle tissues, cardiovascular system and the liver^42^ make it consequently pivotal to define cardiometabolic disease. Importantly, CVD is to this date the main cause of death in NAFLD patients (38% of all mortality^43^) with fibrosis being identified as its most powerful predictor.

Among non-invasive serum markers, AST to Platelet Ratio Index (APRI) has been proposed as a useful score to predict the grade of fibrosis or cirrhosis especially in chronic hepatitis C (CHC)^44^. In this study, we aimed to validate, in a cohort of non-metabolic subjects and MetS patients without a previous CHC diagnosis, the correlation between APRI and CVR and the role of elevated APRI as a marker to predict severe CVR in MetS patients. To define CVR, we used the Framingham Heart Study^45^. Having considered the average age of our study population, as well as the risk factors associated with MetS (diabetes, hypertension, HDL cholesterol), we used the coronary heart disease calculator adjusted for lipids to predict the risk in our cohort to be facing a first cardiovascular event in the coming ten years after first evaluation.

Several studies have clarified the role of WC as predictor for steatohepatitis, fibrosis and insulin resistance^46–48^. To remove any potential biases, we eliminated from our study patients that took antiplatelet or anticoagulant drugs. Combined use of very low-dose antiplatelet plus common anticoagulant has been demonstrated as beneficial in atherosclerotic diseases, including coronary and peripheral artery disease^49^. Antiplatelet could as well alter the APRI result by regulating the platelet count.

For both genders, coronary heart disease (CHD) is the main condition related to CVD morbidity and mortality^50^. It has been demonstrated that the prevalence of CHD is higher in older men, however recent data demonstrated a significant increase of CHD mortality among women in premenopausal age^51^. Lifestyle risk factors play a pivotal role and may vary by race, gender, and ethnicity^52^. Though it started in the Western world, MetS has become rapidly the main lifestyle-related disease across the globe to determine a spike in CVD in both genders^53^. Despite having a higher risk at postmenopausal age, women are naturally protected against cardiovascular disease during reproductive age^54^ compared to age-matched males. This protection is in part attributed to estrogen (E2), even though there are several controversies about the clinical use of exogenous E2 as therapy for CVD due to possible arrhythmia, thrombotic events and, eventually, cancer^55^. Both sexes exhibited common conditions: obesity was frequent among older patients compared to younger subjects^56^.

For the first time, we observed that the role of fibrosis is crucial to alter the estrogen protection in younger females especially with simultaneous diagnosis of MetS: women with elevated APRI reached the same CVR as men even when analyzing the 18–50 years of age range. In this scenario, APRI > 0.5 determines an increase in CVR that is 3 × in MetS patients compared to non-metabolic subjects in both genders, while still causing a significant increase in non-metabolic patients with APRI > 0.5 matched with subjects in similar condition with APRI < 0.5

In conclusion, in the present study we demonstrated the role of APRI score as a valuable predictor for CVR. In a mixed cohort of non-metabolic and MetS subjects, APRI significantly correlates with CVR and determines when > 0.5 a significant increase in CVR in both genders, especially females. This spike in CVR is relatively high in older patients but is significantly higher in younger and premenopausal women, approaching risk values that are usually typical of men. Elevated APRI in patients with MetS diagnosis is related to the highest level of CVR, validating the role of liver fibrosis as a crucial factor to predict CVD in patients that present both conditions. Taken together, our data highlighted the role of APRI as a reliable predictor easy-to-use score for CVR in metabolic patients. Further studies are needed to debate APRI score and CVR in metabolic subjects during dietary and pharmacological treatment.

## Methods

### Study population

Patient recruitments, clinical and biochemical assessment have been consecutively registered from January 2016 to December 2019 in the electronic health register of Metabolic Diseases of Department of Interdisciplinary Medicine, Internal Medicine Division, “Aldo Moro” University of Bari, Italy. This register included 1653 patients affected by metabolic diseases like MetS, diabetes mellitus, hypertension, and dyslipidemias. Among the 1653 patients, 1325 were first evaluations whereas 328 were follow-up observations that were excluded from the study. Then, 43 patients who admitted having abused alcohol in the recent years were excluded from the study, bringing the total population to 1282 patients. Next, we noticed that 30 out of these 1282 patients lacked the value of waist circumference, so they were not included in the evaluation. Then, we detected that blood count was not available for 21 out of the total 1252 patients, thence we removed them from the study population reaching the number of 1231 patients. The diagnosis of Inflammatory Bowel Disease and/or Coeliac disease allowed us to rule out 6 patients more. Indeed, the population under study reached the number of 1225, whose data were free from any analysis restriction (Fig. 1a). The following were considered exclusion criteria: acute heart diseases (cardiac failure, coronary arterial disease, acute arrhythmias), renal and hepatic failure, infections. The subsequent chronic conditions were also considered as exclusion criteria: secondary hypertension, chronic systemic inflammatory diseases, and neoplastic diseases with recent onset (less than 10 years) and/or under chemotherapy. The population included patients with chronic hypertension (also with a chronic condition of hypertensive cardiopathy), chronic diabetes, chronic renal failure, obesity and chronic gastrointestinal diseases (gastroesophageal reflux, chronic gastritis, irritable bowel syndrome). The study protocol was approved by the Ethics Committee of the Azienda Ospedaliero-Universitaria Policlinico di Bari, Italy. The study was conducted in accordance with the ethical principles of the Helsinki Declaration. All patients authorized the present study giving their written informed consent for the use of clinical data.

### Clinical evaluation and anthropometric measurements

Anthropometric parameters (height, body weight, waist circumference) were measured using standardized procedures. Systolic and diastolic blood pressure were determined following three consecutive evaluations. Waist circumference (WC) was measured at the midpoint between the inferior part of the 12th costa and the anterior–superior iliac crests. BMI (body mass index) was calculated as body weight (expressed in kg) divided by squared height expressed in meters (m^2^); conditions of normal weight, overweight and obesity were identified according to BMI values of respectively 20.0–24.9, 25.0–29.9 and 30.0 kg/m^2^. Liver blood tests such as AST (SGOT) and ALT (SGPT) were determined using cut-off values of 37 U/L and 78 U/L, respectively^57^.

Non- invasive liver fibrosis scores (APRI, FIB-4) were determined according to published formulas. APRI score was calculated as AST (IU/l)/platelet count (× 109/l) × 100. The cut-off adopted^58^ in the cited studies were as follows: APRI < 0.5 to identify a fibrosis—free liver, APRI > 0.5 for liver fibrosis and APRI > 1.5 for probable cirrhosis. FIB-4 index was calculated^59^ as age × AST (IU/l)/platelet count (× 109/l) × √ ALT (IU/l). The cut-offs adopted were as followed: FIB-4 < 1.45 for no or moderate fibrosis 1.45 < FIB-4 < 3.25 for moderate fibrosis, FIB-4 > 3.25 for extensive fibrosis or cirrhosis.

The CVR was calculated according to the official FHS calculator for cardiovascular disease^20,45^ in the upcoming 10-years adjusted for lipids and extended to include patients with 18 or more years according to the approved Ethics Committee.

Prediabetes (preDM), Diabetes Mellitus (DM) and Hypertension were defined according to the criteria approved by the International community^60^. For preDM, the criteria were: fasting plasma glucose (FPG) ≥ 100 ≤ 125 mg/dL and HbA1c (percentage of glycosylated hemoglobin) ≥ 5.7% ≤ 6.4%. For DM, the criteria were: HbA1c (percentage of glycosylated haemoglobin) ≥ 6.5%, fasting plasma glucose (FPG) ≥ 126 mg/dL and/or treatment for diabetes. Hypertension was defined as systolic arterial blood pressure (SAP) ≥ 140 mmHg, diastolic arterial blood pressure (DAP) ≥ 90 mmHg and/or treatment with antihypertensive agents. To define dyslipidaemia, HDL cut-off was < 40 mg/dL in men and < 50 mg/dL in women. In addition, the reference value of TG was 150 mg/dL for both genders, whereas a total cholesterol level of ≥ 200 mg/dl was used for the diagnosis of hypercholesterolemia. The diagnosis of MetS was made according to 2006 IDF definition^61^. Thence, the population was classified also considering the presence/absence of each criterion of MetS.

### Biochemical measurements

After overnight fasting, serum was collected for the measurement of standard biochemical markers of glucose and lipid metabolism, liver, renal, thyroid function, and inflammation by standard biochemical methods.

The homeostatic model assessment for insulin resistance (HOMA-IR^62^) was calculated by the following formula (fasting plasma glucose (FPG) × fasting plasma insulin/405).

### Statistical analysis

Descriptive statistical analyses of study sample were performed, and their results were expressed as mean ± standard error of the mean (SEM) and frequencies (%), depending on the nature of variables. Comparisons of socio-demographic and clinical parameters between two groups of interest were carried out with the t-test (for continuous variables) and the Pearson χ^2^ test (for categorical variables). Comparisons between more than two groups were studied through one-way analysis of variance (ANOVA) followed by Bonferroni post-hoc test, where necessary. The correlation between continuous variables was also considered and calculated using Pearson’s Correlation Coefficient (r). P-values lower than 0.05 were regarded as significant. All the analyses were performed using the R statistical environment, version 3.5.2 (The R Foundation for Statistical Computing; Vienna, Austria), NCSS 2007 Statistical Software, version 0.7.10 (NCSS, LLC Company) and GraphPad Prism, version 7.04 (GraphPad Software; San Diego, USA).

## Supplementary Information

Supplementary Table S1.

Supplementary Table S2.

## Abbreviations

ALP: Alkaline phosphatase
ALT: Alanine transaminase
AST: Aspartate transaminase
BMI: Body Mass Index
cIMT: Carotid intima media thickness
DBP: Diastolic blood pressure
ESR: Erythrocyte sedimentation rate
GGT: Gamma-glutamyltransferase
HbA1c: Glycosylated hemoglobin
HDL-C: High-density lipoprotein cholesterol
LDL-C: Low-density lipoprotein cholesterol
SBP: Systolic blood pressure
TC: Total cholesterol
TG: Triglyceride
Hs-CRP: High-sensitivity C reactive protein
WC: Waist circumference

## References

[1] Eckel RH, Grundy SM, Zimmet PZ (2005). The metabolic syndrome. Lancet.

[2] Huang PL (2009). A comprehensive definition for metabolic syndrome. Dis. Model Mech..

[3] Cust AE (2007). Metabolic syndrome, plasma lipid, lipoprotein and glucose levels, and endometrial cancer risk in the European Prospective Investigation into Cancer and Nutrition (EPIC). Endocr. Relat. Cancer.

[4] Libby P, Ridker PM, Hansson GK (2011). Progress and challenges in translating the biology of atherosclerosis. Nature.

[5] Hotamisligil GS (2006). Inflammation and metabolic disorders. Nature.

[6] Satapathy SK, Sanyal AJ (2015). Epidemiology and natural history of nonalcoholic fatty liver disease. Semin. Liver Dis..

[7] ó Hartaigh B (2012). Which leukocyte subsets predict cardiovascular mortality? From the LUdwigshafen RIsk and Cardiovascular Health (LURIC) Study. Atherosclerosis.

[8] Bhat T (2013). Neutrophil to lymphocyte ratio and cardiovascular diseases: A review. Expert. Rev. Cardiovasc. Ther..

[9] Buyukkaya E (2014). Correlation of neutrophil to lymphocyte ratio with the presence and severity of metabolic syndrome. Clin. Appl. Thromb. Hemost..

[10] Gary T (2013). Platelet-to-lymphocyte ratio: A novel marker for critical limb ischemia in peripheral arterial occlusive disease patients. PLoS ONE.

[11] Balta S, Ozturk C (2015). The platelet-lymphocyte ratio: A simple, inexpensive and rapid prognostic marker for cardiovascular events. Platelets.

[12] Vahit D, Akboga MK, Samet Y, Huseyin E (2017). Assessment of monocyte to high density lipoprotein cholesterol ratio and lymphocyte-to-monocyte ratio in patients with metabolic syndrome. Biomark. Med..

[13] Gong S (2018). Association of lymphocyte to monocyte ratio with severity of coronary artery disease. Medicine.

[14] Akboga MK (2016). Platelet to lymphocyte ratio as a novel indicator of inflammation is correlated with the severity of metabolic syndrome: A single center large-scale study. Platelets.

[15] Sun W (2016). Comparison of FIB-4 index, NAFLD fibrosis score and BARD score for prediction of advanced fibrosis in adult patients with non-alcoholic fatty liver disease: A meta-analysis study. Hepatol. Res..

[16] Ganjali S (2018). Monocyte-to-HDL-cholesterol ratio as a prognostic marker in cardiovascular diseases. J. Cell Physiol..

[17] Uslu AU (2018). Evaluation of monocyte to high-density lipoprotein cholesterol ratio in the presence and severity of metabolic syndrome. Clin. Appl. Thromb. Hemost..

[18] Yilmaz Y (2011). Noninvasive assessment of liver fibrosis with the aspartate transaminase to platelet ratio index (APRI): Usefulness in patients with chronic liver disease: APRI in chronic liver disease. Hepat. Mon..

[19] Lai M, Afdhal NH (2019). Liver fibrosis determination. Gastroenterol. Clin. N. Am..

[20] Mahmood SS, Levy D, Vasan RS, Wang TJ (2014). The Framingham Heart Study and the epidemiology of cardiovascular disease: A historical perspective. Lancet.

[21] Weir, C. B. & Jan, A. BMI Classification Percentile And Cut Off Points. *StatPearls* (2020). 31082114

[22] VanWagner LB (2015). Association of nonalcoholic fatty liver disease with subclinical myocardial remodeling and dysfunction: A population-based study. Hepatology.

[23] Fotbolcu H (2010). Impairment of the left ventricular systolic and diastolic function in patients with non-alcoholic fatty liver disease. Cardiol. J..

[24] Madan SA, John F, Pyrsopoulos N, Pitchumoni CS (2015). Nonalcoholic fatty liver disease and carotid artery atherosclerosis in children and adults: A meta-analysis. Eur. J. Gastroenterol. Hepatol..

[25] Chalasani N, Deeg MA, Crabb DW (2004). Systemic levels of lipid peroxidation and its metabolic and dietary correlates in patients with nonalcoholic steatohepatitis. Am. J. Gastroenterol..

[26] Madan K, Bhardwaj P, Thareja S, Gupta SD, Saraya A (2006). Oxidant stress and antioxidant status among patients with nonalcoholic fatty liver disease (NAFLD). J. Clin. Gastroenterol..

[27] Alkhouri N (2010). The inflamed liver and atherosclerosis: A link between histologic severity of nonalcoholic fatty liver disease and increased cardiovascular risk. Dig. Dis. Sci..

[28] Siddiqui MS (2015). Severity of nonalcoholic fatty liver disease and progression to cirrhosis are associated with atherogenic lipoprotein profile. Clin. Gastroenterol. Hepatol..

[29] Francque SM, van der Graaff D, Kwanten WJ (2016). Non-alcoholic fatty liver disease and cardiovascular risk: Pathophysiological mechanisms and implications. J. Hepatol..

[30] Ekstedt M (2015). Fibrosis stage is the strongest predictor for disease-specific mortality in NAFLD after up to 33 years of follow-up. Hepatology.

[31] Soderberg C (2010). Decreased survival of subjects with elevated liver function tests during a 28-year follow-up. Hepatology.

[32] Lazo M (2011). Non-alcoholic fatty liver disease and mortality among US adults: Prospective cohort study. BMJ.

[33] Stepanova M, Younossi ZM (2012). Independent association between nonalcoholic fatty liver disease and cardiovascular disease in the US population. Clin. Gastroenterol. Hepatol..

[34] European Association for the Study of the, L., European Association for the Study of, D., European Association for the Study of, O (2016). EASL-EASD-EASO Clinical Practice Guidelines for the management of non-alcoholic fatty liver disease. J. Hepatol..

[35] Tripodi A (2014). Procoagulant imbalance in patients with non-alcoholic fatty liver disease. J. Hepatol..

[36] Yu KJ, Zhang MJ, Li Y, Wang RT (2014). Increased whole blood viscosity associated with arterial stiffness in patients with non-alcoholic fatty liver disease. J. Gastroenterol. Hepatol..

[37] Zhao HY (2015). Elevated whole blood viscosity is associated with insulin resistance and non-alcoholic fatty liver. Clin. Endocrinol..

[38] Ballestri S (2021). Liver fibrosis biomarkers accurately exclude advanced fibrosis and are associated with higher cardiovascular risk scores in patients with NAFLD or viral chronic liver disease. Diagnostics.

[39] Rockey DC (2009). Liver biopsy. Hepatology.

[40] Chen Q (2020). Association between liver fibrosis scores and the risk of mortality among patients with coronary artery disease. Atherosclerosis.

[41] Han HS, Kang G, Kim JS, Choi BH, Koo SH (2016). Regulation of glucose metabolism from a liver-centric perspective. Exp. Mol. Med..

[42] Tilg H, Moschen AR (2010). Evolution of inflammation in nonalcoholic fatty liver disease: The multiple parallel hits hypothesis. Hepatology.

[43] Angulo P (2015). Liver fibrosis, but no other histologic features, is associated with long-term outcomes of patients with nonalcoholic fatty liver disease. Gastroenterology.

[44] Wong S, Huynh D, Zhang F, Nguyen NQ (2017). Use of aspartate aminotransferase to platelet ratio to reduce the need for FibroScan in the evaluation of liver fibrosis. World J. Hepatol..

[45] D'Agostino RB (2008). General cardiovascular risk profile for use in primary care: The Framingham Heart Study. Circulation.

[46] Pischon T (2008). General and abdominal adiposity and risk of death in Europe. N. Engl. J. Med..

[47] Campos-Nonato I, Hernandez L, Barquera S (2017). Effect of a high-protein diet versus standard-protein diet on weight loss and biomarkers of metabolic syndrome: A randomized clinical trial. Obes. Facts.

[48] Marchesini G (2003). Nonalcoholic fatty liver, steatohepatitis, and the metabolic syndrome. Hepatology.

[49] De Caterina R (2009). The current role of anticoagulants in cardiovascular medicine. J. Cardiovasc. Med..

[50] Gerdts E, Regitz-Zagrosek V (2019). Sex differences in cardiometabolic disorders. Nat. Med..

[51] Mosca L, Barrett-Connor E, Wenger NK (2011). Sex/gender differences in cardiovascular disease prevention: What a difference a decade makes. Circulation.

[52] Herd P, Karraker A, Friedman E (2012). The social patterns of a biological risk factor for disease: Race, gender, socioeconomic position, and C-reactive protein. J. Gerontol. B Psychol. Sci. Soc. Sci..

[53] Saklayen MG (2018). The global epidemic of the metabolic syndrome. Curr. Hypertens. Rep..

[54] Iorga A (2017). The protective role of estrogen and estrogen receptors in cardiovascular disease and the controversial use of estrogen therapy. Biol. Sex Differ..

[55] Naftolin F, Friedenthal J, Nachtigall R, Nachtigall L (2019). Cardiovascular health and the menopausal woman: The role of estrogen and when to begin and end hormone treatment. F1000Research.

[56] Osher E, Stern N (2009). Obesity in elderly subjects: In sheep's clothing perhaps, but still a wolf!. Diabetes Care.

[57] Battaglia S (2020). Gender, BMI and fasting hyperglycaemia influence monocyte to-HDL ratio (MHR) index in metabolic subjects. PLoS ONE.

[58] Jain P, Sharda M, Bauddh NK, Ajmera D (2020). APRI score: A screening marker of hepatic fibrosis in NAFLD patients. J. Assoc. Physicians India.

[59] Mallet V (2019). FIB-4 index to rule-out advanced liver fibrosis in NAFLD patients. Presse Med..

[60] Guthrie RA, Guthrie DW (2004). Pathophysiology of diabetes mellitus. Crit. Care Nurs. Q..

[61] Alberti KG, Zimmet P, Shaw J (2006). Metabolic syndrome—a new world-wide definition. A Consensus Statement from the International Diabetes Federation. Diabet. Med..

[62] Matthews DR (1985). Homeostasis model assessment: Insulin resistance and beta-cell function from fasting plasma glucose and insulin concentrations in man. Diabetologia.

