# Supplementary material for: AST to Platelet Ratio Index (APRI) is an easy-to-use predictor score for cardiovascular risk in metabolic subjects
Source: Sci Rep. 2021 Jul 21;11:14834. doi: 10.1038/s41598-021-94277-3 (PMC8295377; doi:10.1038/s41598-021-94277-3)
Supplement: Supplementary file 1 — Supplementary Table S1. [file 41598_2021_94277_MOESM1_ESM.docx]

**Supplementary Table 1. Clinical characterization of the study population.**

| **Clinical variable** | **MetS NO** | **MetS YES** | **p-value** |
| --- | --- | --- | --- |
| n (M:F) | 685 (336:349) | 540 (287:263) | - |
| Age (years) | 52.58±0.59 | 61.31± 0.52 | <0.05 |
| Weight (Kg) | 70.2±0.59 | 84.8± 0.76 | <0.05 |
| Waist circumference (cm) | 91.5±0.51 | 106.7± 0.58 | <0.05 |
| BMI (Kg/m^2^) | 25.2±0.19 | 30.6± 0.24 | <0.05 |
| Sistolic blood pressure (mmHg) | 122.0±0.58 | 134.4±0.73 | <0.05 |
| Diastolic blood pressure (mmHg) | 76.3±0.37 | 80.9±0.44 | <0.05 |
| Platelet count (10^6/μL) | 239.0±2.36 | 233.7±2.38 | NS |
| Hemoglobin (g/dl) | 13.89±0.09 | 14.05±0.10 | NS |
| WBC (10^3^/µl) | 6.03±0.07 | 6.98±0.08 | <0.01 |
| Monocytes (%) | 6.33±0.07 | 6.36±0.07 | NS |
| Lymphocytes (%) | 32.97±0.30 | 31.15±0.33 | NS |
| Neutrophils (%) | 57.45±0.32 | 59.26±0.34 | NS |
| Basophils (%) | 0.54±0.01 | 0.55±0.02 | NS |
| Eosinophils (%) | 2.76±0.07 | 2.75±0.07 | NS |
| Glucose (mg/dl) | 89.6±0.62 | 117.9±1.79 | <0.05 |
| HbA1c (mmol/mol) | 37.5±0.45 | 46.6±0.71 | <0.05 |
| Total cholesterol (mg/dl) | 186.9±1.40 | 178.2±2.04 | NS |
| HDL-c (mg/dl) | 59.9±0.56 | 46.2±0.57 | <0.05 |
| LDL-c (mg/dl) | 108.6±1.27 | 98.9±1.55 | NS |
| TG (mg/dl) | 92.7±1.48 | 160.8±3.86 | <0.05 |
| AST (U/I) | 21.6±0.29 | 24.5±0.52 | NS |
| ALT (U/I) | 28.1±0.51 | 35.1±1.01 | <0.05 |
| ALP (U/I) | 67.34±0.98 | 73.54±1.28 | <0.05 |
| GGT (U/I) | 29.2±1.04 | 40.4±1.82 | <0.05 |
| Ferritin (ng/ml) | 98.9±5.32 | 125.6±7.78 | <0.05 |
| Iron (ug/dl) | 85.68±2.32 | 81,3±2.78 | <0.05 |
| Creatinine (mg/dl) | 0.85±0.01 | 0.77±0.01 | <0.05 |
| Uric acid (mg/dl) | 48.67±0.19 | 58.08±0.35 | NS |
| Total protein (g/dl) | 7.24±0.03 | 7.32±0.03 | NS |
| Albumin (g/dl) | 4.58±0.81 | 4.95±0.42 | NS |
| ESR (mm/h) | 14.02±0.51 | 20.12±0.84 | NS |
| Hs-CRP (mg/l) | 3.68±0.14 | 4.51±0.26 | <0.05 |
| TSH (mUI/L) | 2.02±0.15 | 2.13±0.11 | NS |
| FT3 (pg/ml) | 2.81±0.03 | 2.77±0.03 | NS |
| FT4 (ng/dl) | 1.02±0.11 | 1.05±0.13 | NS |
| Ab anti TG (UI/ml) | 42.74±11.03 | 77.12±50.82 | NS |
| Ab anti TPO (UI/ml) | 396.11±143.76 | 194.55±158.47 | <0.05 |
| Cardiovascular risk (Framingham) | 11.4±0.49 | 28.5±0.81 | <0.05 |
| FIB-4 score | 1.1±0.02 | 1.3±0.03 | <0.01 |
| APRI score | 0.3±0.01 | 0.4±0.01 | <0.01 |
|  |  |  |  |

Data are presented as mean ± SEM (standard error of the mean). Abbreviations: Body Mass Index, BMI; Waist Circumference, WC; systolic blood pressure, SBP; diastolic blood pressure, DBP; glycosylated hemoglobin, HbA1c; total cholesterol, TC; high-density lipoprotein cholesterol, HDL-C; low-density lipoprotein cholesterol, LDL-C; triglyceride, TG; aspartate transaminase, AST; alanine transaminase, ALT; alkaline phosphatase, ALP; gamma-glutamyltransferase, GGT; erythrocyte sedimentation rate, ESR; high-sensitivity C reactive protein Hs-CRP.
