# Supplementary material for: AST to Platelet Ratio Index (APRI) is an easy-to-use predictor score for cardiovascular risk in metabolic subjects
Source: Sci Rep. 2021 Jul 21;11:14834. doi: 10.1038/s41598-021-94277-3 (PMC8295377; doi:10.1038/s41598-021-94277-3)
Supplement: Supplementary file 2 — Supplementary Table S2. [file 41598_2021_94277_MOESM2_ESM.docx]

**Supplementary Table 2. Clinical comparison between healthy and MetS subjects with normal and elevated APRI score in the study population.**

|  | **MetS NO** | | **MetS YES** | |  |
| --- | --- | --- | --- | --- | --- |
| **Clinical variable** | **APRI <0.5** | **APRI >0.5** | **APRI <0.5** | **APRI >0.5** | **p-value** |
| n (M:F) | 587 (283:304) | 98 (53:45) | 406 (216:200) | 134 (71:63) | - |
| Age (years) | 51.65±0.63 | 57.89±1.90 | 61.70± 0.57^a^ | 62.03±1.19 | <0.05 |
| Weight (Kg) | 69.42±0.65 | 73.82±1.94 | 84.04± 0.81^a^ | 87.11±1.91 | <0.05 |
| Waist circumference (cm) | 90.97±0.54 | 94.45±1.83 | 106.65± 0.60^a,b^ | 106.53±1.41^a^ | <0.01 |
| BMI (Kg/m^2^) | 25.04±0.19 | 26.05±0.61 | 30.60± 0.24 | 30.52±0.53 | NS |
| Sistolic blood pressure (mmHg) | 121.01±0.60 | 124.8±2.13 | 134.52±0.85^a^ | 134.39±1.57^a^ | <0.05 |
| Diastolic blood pressure (mmHg) | 76.19±0.40 | 76.42±1.17 | 80.70±0.51 | 81.35±1.11 | NS |
| Platelet count (10^6/μL) | 245.04±2.48 | 170.72±5.30 | 247.30±2.92 | 178.98±4.45 | <0.05 |
| Hemoglobin (g/dl) | 13.86±0.10 | 14.07±0.22 | 13.92±1.42 | 14.15±0.15 | NS |
| WBC (103/µl) | 6.04±0.07 | 5.58±0.21 | 7.14±1.35 | 6.30±0.17 | NS |
| Monocytes (%) | 6.27±0.08 | 6.79±0.24 | 6.25±0.07 | 6.76±0.17 | NS |
| Lymphocytes (%) | 32.97±0.32 | 32.97±1.03 | 30.75±0.30 | 32.97±0.84 | NS |
| Neutrophils (%) | 57.56±0.36 | 56.42±1.18 | 59.92±0.34 | 57.09±0.74 | NS |
| Basophils (%) | 0.54±0.02 | 0.5±0.07 | 0.55±0.01 | 0.58±0.02 | NS |
| Eosinophils (%) | 2.75±0.17 | 2.86±0.22 | 2.74±0.85 | 2.78±0.18 | NS |
| Glucose (mg/dl) | 89.36±0.67 | 92.46±2.43 | 118.64±2.13^a,b^ | 119.52±3.90^a,b^ | <0.05 |
| HbA1c (mmol/mol) | 37.49±0.40 | 37.81±1.65 | 46.69±0.80 | 46.81±1.54 | <0.05 |
| Total cholesterol (mg/dl) | 188.32±1.50 | 175.07±5.51 | 176.38±2.20 | 181.18±4.29 | NS |
| HDL-c (mg/dl) | 60.71±0.60 | 54.69±2.33 | 47.12±0.65^a,b^ | 44.42±1.31^a,b^ | <0.01 |
| LDL-c (mg/dl) | 109.48±1.35 | 99.87±4.55 | 98.19±1.74 | 99.22±3.73 | NS |
| TG (mg/dl) | 91.81±1.62 | 96.11±3.93 | 157.30±4.45^a,b^ | 165.1±8.30^a,b^ | <0.05 |
| AST (U/I) | 20.09±0.26 | 34.62±1.31^a^ | 20.51±0.27 | 40.83±1.53^a^ | <0.01 |
| ALT (U/I) | 26.40±0.40 | 43.73±3.01^a,c^ | 29.39±0.53 | 58.24±3.73^a,c^ | <0.05 |
| ALP (U/I) | 66.12±1.01 | 75.15±3.37 | 73.68±1.45 | 73.26±2.74 | <0.05 |
| GGT (U/I) | 27.20±0.94 | 47.40±6.34^a^ | 35.01±1.82 | 62.07±5.05^a^ | <0.01 |
| Ferritin (ng/ml) | 89.77±4.74 | 160.74±19.31^a,c^ | 108.45±7.31^a^ | 190.71±19.53^a,c^ | <0.01 |
| Iron (ug/dl) | 84.22±2.40 | 93.81±6.48 | 83.02±3.31 | 75.52±4.50b | <0.05 |
| Creatinine (mg/dl) | 0.80±1.50 | 0.85±0.02 | 0.86±1.29 | 0.87±2.21 | NS |
| Uric acid (mg/dl) | 47.89±0.20 | 57.74±0.80 | 57.52±0.42 | 60.32±0.77^a^ | <0.05 |
| Total protein (g/dl) | 7.24±1.41 | 7.22±5.05 | 7.28±0.03 | 7.41±5.21 | NS |
| Albumin (g/dl) | 4.61±0.21 | 4.43±3.12 | 5.13±0.55 | 4.37±0.17 | NS |
| ESR (mm/h) | 14.62±0.54 | 14.38±1.81 | 20.30±0.90^a,b^ | 18.80±1.64 | NS |
| Hs-CRP (mg/l) | 3.67±0.15 | 3.77±0.46 | 4.77±0.32 | 4.70±0.21 | NS |
| TSH (mUI/L) | 1.83±0.11 | 2.03±0.19 | 2.07±0.13 | 1.96±0.22 | NS |
| FT3 (pg/ml) | 2.81±2.01 | 2.80±7.01 | 2.77±2.42 | 2.80±6.01 | NS |
| FT4 (ng/dl) | 1.03±1.25 | 0.99±1.01 | 1.05±1.15 | 1.07±3.01 | NS |
| Ab anti TG (UI/ml) | 42.75±11.03 | 42.69±27.31 | 95.42±65.41 | 15.37±2.04 | NS |
| Ab anti TPO (UI/ml) | 441.71±161.54 | 38.39±16.43 | 248.17±206.87 | 19.09±3.05 | NS |
| Cardiovascular risk (Framingham) | 10.01±0.40 | 25.01±2.43^a^ | 25.95±0.84^a^ | 41.67±2.05^a,b,c^ | <0.01 |
| FIB-4 score | 0.91±0.02 | 2.01±0.16^a^ | 1.0±0.02 | 2.24±0.11^a^ | <0.05 |
| APRI score | 0.27±0.04 | 0.68±0.03^a,c^ | 0.30±0.01 | 0.76±0.07^a,c^ | <0.01 |

Data are presented as mean ± SEM (standard error of the mean). Abbreviations: Body Mass Index, BMI; Waist Circumference, WC; systolic blood pressure, SBP; diastolic blood pressure, DBP; total cholesterol, TC; triglyceride, TG; high-density lipoprotein cholesterol, HDL-C; low-density lipoprotein cholesterol, LDL-C; glycosylated hemoglobin, HbA1c; high-sensitivity C reactive protein Hs-CRP; erythrocyte sedimentation rate, ESR; gamma-glutamyltransferase, GGT; aspartate transaminase, AST; alanine transaminase, ALT; alkaline phosphatase, ALP; ^a^ indicates statistical significance compared to HEALTHY with APRI<0.5 group , ^b^ indicates statistical significance compared to HEALTHY with APRI>0.5 group, ^c^ indicates statistical significance to MetS with APRI<0.5 group.
